# Opportunities to cultivate trust through communication behaviors

**DOI:** 10.3389/fgene.2026.1847245

**Published:** 2026-09-03

**Authors:** Mary Carol Barks, Claire Yballa, Alexandra Linker, Chau Amoeni, Gabrielle Cua, Malley Craig, Bridget Hein, Thomas May

**Affiliations:** 1 Elson S. Floyd College of Medicine, Washington State University, Spokane, WA, United States; 2 Graduate Division, University of California, San Francisco, CA, United States; 3 University of California, Los Angeles, CA, United States; 4 University of South Florida, Tampa, FL, United States

**Keywords:** social implications, biobanks ethics, ELSI (ethical, legal, trust)

## Abstract

Sound scientific sample diversity will require attention to cultivating trust among populations underrepresented as biobank participants. While no particular group is the focus of this report, the observations presented may be particularly helpful for cultivating trust necessary for enrollment of underrepresented populations. Community engagement strategies indicate that such trust is built through relationships extending across the spectrum of biomedicine rather than focused exclusively on a particular research project. Central to these relationships are communication behaviors reflected in both research and clinical encounters. This ‘brief research report’ presents preliminary clinical research findings from a pilot analysis of return of genetic results conversations relevant to the cultivation of trust.

## 1 Introduction

Willingness to participate in biomedical research of any type requires trust, and this is nowhere more true than in biobank research, where subjects’ biological samples are held for unspecified future research. This brief report will focus on missed opportunities to cultivate trust needed to secure participation in biobanks and data repositories. Elsewhere, author T.M. has argued that life experiences of many disadvantaged groups can result in suspicion of research repositories where the potential for problematic use of data and materials beyond original consented-to purposes can discourage participation in research intending to contribute to repositories (May, 2020). Historical examples of deleterious misuse of “re-purposed” research data and materials are plentiful, ranging from harms to groups based on race (e.g., Havasupai genetic research) (Mello and Wolf, 2010) to disabilities (e.g., Nazi research on people with disabilities designed to be used for the creation of a “master race”) (Engber, 2004).

Good science requires not only participation of subjects in general, but from diverse populations, as articulated and justified in the target enrollment characteristics of regional (AGHI (May et al., 2020)), National (All of Us (NIH, 2026)), and International (The European Genome-phenome Archive (EG A, 2026); H3Africa (H3Africa, 2011)) data initiatives. Each of these reflect programs widely recognized as designed to “bridge gaps” in data diversity in the name of better science (Omotoso et al., 2022; Corpas et al., 2025). While no particular group is the focus of this report, the observations presented may be particularly helpful for cultivating trust necessary for enrollment of underrepresented populations.

Overcoming gaps in sample diversity needed for good science utilizing biobanks will require significant attention to cultivating trust. The most promising strategies to facilitate trust and participation in research involve building long-term relationships that extend beyond specific research projects (May et al., 2021). Trust and familiarity develop over time and across contexts, with negative experiences in one setting influencing presumptions of trust in others. It is for this reason that community engagement approaches have emphasized that building longstanding relationships and extending these to concerns outside the purview of research is key; the Community Based Participatory Research (CBPR) approach in particular emphasizes the incorporation of engaged communities into each stage of projects, including the formulation of purposes and aims, to ensure the research acknowledges and reflects that community’s specific interests, concerns, and social circumstances (Israel et al., 2012; Minkler and Wallerstein, 2011).

In addition, our team’s own prior research identified health communication that acknowledges life experiences and circumstances as major factors in establishing both trust and a willingness to participate in research; examples include minority participants’ greater distrust of medical literature and more frequent concern to receive medical information from members of the same race (Smith et al., 2025; Barks and May, 2026). Thus, if we are to address underrepresentation of disadvantaged groups among biobank participants, there is a present need for greater attention to communication behaviors that acknowledge and are sensitive to the unique lived experiences of disadvantaged individuals in clinical conversations. Because trust (and distrust) extend over multiple contexts, this attention must as well.

In sum, it is essential to study the ways in which medical providers communicate across the spectrum of both research and clinical medicine. Our team’s current research is aimed toward analyzing these very communication behaviors present in conversations where clinicians discuss genetic test results with parents of critically ill infants. Examination of communication behaviors in these clinical contexts will be essential to identify the ways in which subtle behaviors—especially communication behaviors that acknowledge and respect the lived experience of underrepresented groups—either threaten to undermine trust or fail to cultivate trust between the medical community and communities that are clinically underserved and underrepresented in research (particularly research involving repositories, for reasons discussed above).

## 2 Methods

Our analysis aims to characterize both the content and context of clinical return-of-results conversations in terms of understanding how specific communication behaviors may serve to promote and undermine trust in different populations. Specifically, our study involves secondary analysis of a larger study where clinicians explained the results of genetic tests to parents of infants admitted to the neonatal intensive care unit (NICU). Approximately 400 of these conversations were audio recorded under the NIH “SouthSeq” IRB protocol (UAB IRB-300000328). Infants admitted to participating NICUs in Alabama, Kentucky, Louisiana, and Mississippi with a suspected genetic condition were eligible to enroll. Once enrolled, each infant received whole genome sequencing (WGS); genetic results were returned either in-person or by phone to each child’s parents by either a genetic counselor or non-genetics medical provider who underwent brief training in effective genetic return of results (East et al., 2022).

For our preliminary investigation, we transcribed a “pilot set” of 40 recorded return of result conversations for 38 enrolled infants with positive WGS results. Secondary analysis of these transcripts has been conducted under separate review approved by the Washington State University Institutional Review Board (IRB-20464-001). These “pilot” transcripts included infants from families reflecting several demographic features and represent multiple results returned to infants of Black and White parents, parents living in rural and metropolitan locations, and results returned by genetic counselors and by neonatologists.

While our analysis to characterize the content and context of return of result conversations remains at a very preliminary stage, some themes have begun to emerge that are relevant to the issues that are the focus of this brief research report. In particular, we have identified several distinct, reoccurring missed opportunities for clinicians to cultivate trust through communication behaviors. These missed communication opportunities would extend the focus of conversation beyond the immediate medical context to broader humanistic concerns that community engagement approaches have long emphasized as important to establish trust, as discussed in the introduction.

NVivo qualitative software was used to organize and analyze transcripts for identification and occurrence of themes (NVivo, 2025). Transcripts were read and coded by seven of the coauthors for a variety of potential purposes. The observations presented here, while not coded for intentionally, have nonetheless emerged as missed opportunities that may warrant more in-depth study in terms of potential strategies to address inadequate sample diversity needed for good science.

## 3 Results

### 3.1 Infant, parent, and conversation characteristics

Forty genetic return of result conversations were analyzed for 38 infants; two infants in our sample had a second conversation where families received updated results following an initial report of genetic findings. One conversation (n = 1, 3%) occurred following the child’s death (See Table 1).

**TABLE 1:** Conversation, infant, and parent characteristics.

Characteristic	n (%)	
Conversation characteristics, n = 40		
Clinician type		
Genetic counselor	19	(47)
Non-genetics provider	21	(53)
Method of return		
In-person	13	(Ahir-Knight and Godfrey, 2026)
Phone	27	(68)
Infant characteristics, n = 38		
Underserved^a^	30	(79)
Low income	13	(34)
Non-white	23	(61)
Rural^b^	7	(Labrie et al., 2021)
Parent characteristics, n = 38		
Race and ethnicity^a^		
Black	20	(53)
Hispanic	2	(NIH, 2026)
Other	1	(Engber, 2004)
White	22	(58)

^a^ Participants could represent or select more than one category.

^b^ Information on the parents’ and infant’s residence was available for n = 26 infants.

Categories of race for this secondary analysis were defined by the original CSER/Southseq protocol from which this data was collected. Under that protocol, race is defined using standardized self-report demographic categories (recognizing the lack of universal definitions) (CSER Consortium Shared Dataset Documentation, 2023). Although we provide demographic information in the interests of full disclosure of our pilot data sample, this brief report is focused on observations emerging from broad initial categorization of data, rather than an analysis related to the specific aims of the pilot study which seeks to identify differences in communication behaviors between demographic categories. The absence of conversational elements identified are common across the demographic categories, and presented as an observation emerging from our early, broad assessments of the data as a whole, rather than reflective of the pilot study’s larger purpose of identifying differential communication behaviors. Thus, the provision of demographics is informational, rather than part of the relevant assessment.

#### 3.1.1 Results of initial coding

Our analysis of coding is at a very preliminary stage and focuses primarily on identification of areas that merit more in-depth study and greater sample analysis. These broad, initial coding categories are still being refined. Each transcript was independently coded by at least two authors; differences in coding were discussed and reconciled to produce finalized coding for each transcript. While this analysis is too preliminary for a comprehensive report, several things relevant as context to our focus here merit a brief summary. It is important to note that these are meant as context and not findings. In fact, even our codebook is still evolving, and so a report on the findings of the pilot study itself is not yet feasible.

##### 3.1.1.1 A focus on core task of genetic testing

Our preliminary analysis did not identify significant concerns with providers’ coverage of the core tasks of genetic return of results. The focus of conversations generally included discussions of the nature of genetic findings and their relationship to diagnosis and prognosis, which were the core aims of testing under the SouthSeq protocol (East et al., 2022).

##### 3.1.1.2 Describing prognosis

Our coding showed clinicians discussed with parents how the genetic finding would impact the future health of the child in the vast majority (n = 36, 90%) of return of result conversations. Because infants underwent WGS testing based on the presence of symptoms indicative of a genetic condition, these discussions often included predictions on whether the symptoms consistent with their genetic diagnosis would continue, change, or resolve. Clinicians also informed parents of the potential for new challenges to occur based on these reported genetic findings. Clinicians often described a range of possible health issues that a child may experience, as expressed by one genetic counselor:

“People who have Coffin-Siris syndrome have different kinds of facial features, differences in their limbs sometimes. They can have some brain differences. They can have low tone and seizures and learning difficulties.”

Many predictions were characterized by uncertainty in terms of the likelihood a specific health issue would occur and its level of severity. For example, a neonatologist shares their uncertainty about the severity of a child’s expected developmental delay:

“*What are the things that is associated with this condition is a little bit of a delayed development and difficulty in achieving milestones and so on as they grow older. Now, some people have a very severe delay in that. Some kids probably have a milder delay in that. We will never be able to tell which of those she has until she grows older and we follow her and see how she does.”*

##### 3.1.1.3 Explaining inheritance

Nearly every (n = 36, 90%) return of result conversation in our sample included a discussion about inheritance. These conversations included descriptions of how inheritance works (for example, whether the genetic change was dominant or recessive) and whether the genetic change was inherited from the parent, *de novo*, or of unknown in origin. A neonatologist explains how recessive genetic changes from the parents are the reason for their son’s symptoms in the below example:

The changes that they found in you and mom, each in one gene of your blood, were in the WDPCP gene that [child] has. These changes are thought to be recessive, which means that you need two gene changes together to cause the problem. You and mom are just carriers for these gene changes, but when [child] has two different abnormal genes, that’s why he has the symptoms of that gene change.

Clinicians also explained inheritance in terms of risk to other family members, including the risk of genetic differences affecting any future pregnancies of the parents or child. In several discussions, clinicians and parents discussed how a relative’s symptoms may be explained by the reported findings. Clinicians speculated whether the patient’s genetic differences were relevant to a sibling’s cause of death in two conversations.

#### 3.1.2 Missed opportunities to cultivate trust

Clinicians describing prognosis in terms of the potential challenges a child may experience and explaining the role of inheritance are to be expected and are indeed core components of informing parents about genetic findings during return-of-results conversations. At the same time, these conversations are part of the broader interactive patient-provider-relationship, and as such merit attention in terms of their potential role in establishing trust.

While we did not intentionally code for missed opportunities to cultivate trust as a communication behavior, it emerged to authors that the types of “life circumstances” and “lived experience” conversations emphasized by community engagement literature were scarce. Because our own prior research (discussed above) has identified these concerns as important components of trust, we believe that the communication behaviors observed in the pilot set of transcripts we analyzed warrant consideration as potential missed opportunities to cultivate trust. Below we identify and describe three missed opportunities for clinicians to cultivate trust during return of results conversations with NICU parents.

##### 3.1.2.1 Acknowledging lived experience

Despite the prevalence of prognostic information discussed within our sample, these discussions about the future were largely comprised of factual and unbalanced expectations for the child. An illustrative example includes a genetic counselor’s response to a mother asking what to expect for her child’s quality of life:

“Quality of life, um, that's really hard to answer because it does seem like that's been extremely varied from child to child. So, um, you know, it really depends on what other features they're born with. Because, you know, sometimes babies with this condition, if they were born with a complex heart defect, and on top of the other things, they're having to have multiple open-heart surgeries as infants, that's going to slow them down even more.”

The clinician’s response assesses quality of life strictly in terms of the infant’s medical condition and course, based on the likelihood and impact of surgical interventions, and misses an opportunity to explore deeper quality of life issues that may be of concern to the parents. Before responding to this question, the clinician may have first assessed how the child’s mother personally defines “quality of life” to ensure that the information presented aligns with her definition and likely concerns.

Only one return of result conversation (n = 1, 3%) referenced information about the lived experience of children with the same or similar conditions, and even this conversation failed to fully engage the topic. In this single conversation, the genetic counselor encouraged the infant’s parents to visit a website that included “*stories from like fifteen or twenty different kids, and you can see their pictures and kind of compare what they’re doing and what their days look like just for sort of comparison and information sake*.”

##### 3.1.2.2 Addressing parental guilt

Only 17% of transcripts (n = 6) that discussed inheritance included clinicians assuring parents that they were not at fault for their child’s illness. The following example, where a neonatologist confirms that the child’s genetic change was reported in samples from both the mother and father, represents a missed opportunity to address parent guilt during inheritance discussions:

“So we would consider that both of y'all are carrying a single one of these genetic changes in your NPHS1 gene. Now, with him getting two of these changes, one from mom and one from dad, that is the cause of his congenital nephrotic syndrome, okay? So he has two copies of this gene that just are not doing the job that they're supposed to be doing, and that's why his kidneys are having such a hard time.”

Here, the clinician states twice that the son’s kidney issues result from each parent passing on “a single one of these genetic changes.” Confirming that a child’s genetic differences were inherited from one or both parents presents an important opportunity for the clinician to clarify that the parents are not at fault. Addressing parental guilt may be an especially important aspect of these discussions considering that it is common for NICU parents to blame themselves for their child’s health condition, independent of genetic inheritance. The clinician may also have normalized these feelings of guilt, as illustrated in a conversation where parental guilt was addressed by a genetic counselor for a mother of an infant with a *de novo* genetic finding:

Genetic Counselor: “*So sometimes these genetic mutations just happen randomly really, really early in the baby’s development, and it was nothing that you did to cause it. It was just a completely random thing that happened*.”

Mother: “*Yeah, actually, I was kind of worried, you know, because I took good care of my pregnancy; and I was kind of blaming myself for that.*”

Genetic Counselor: “*Yeah, no. It’s normal to feel that way, but it’s really important for you to know that you did nothing wrong, this was out of your control, and not to worry about that. There is nothing you could have done to prevent this*.”

##### 3.1.2.3 Assessing accessibility of care and resources

Few conversations included a focus on the broad social life circumstances and challenges faced by the infant’s family, either pertinent to the day-to-day care of the child or the wellbeing of the child’s family, in this preliminary analysis of conversations. Transcripts lacked explicit inquiries to identify accessibility to health services and the presence of family support resources. Discussions around socioeconomic concerns including income, secure housing, and transportation were also neglected topics in our analysis of recorded conversations.

For example, when one clinician asked a mother where to mail the report of her child’s genetic findings, the mother replied that the results would need to be mailed to the home of a relative “*because we kind of got rid of our house and everything while we were at the hospital*.” Instead of responding to this information by investigating the family’s current living situation, including whether they had housing and transportation that allowed their child to receive adequate care, the clinician proceeded by confirming the new mailing address without asking follow-up questions at a later point in the conversation. By not inquiring about the circumstances of this event, the clinician missed the opportunity to connect the infant’s family with potentially needed resources to address socioeconomic challenges.

## 4 Discussion

Although a focus on factual information relevant to prognosis and inheritance pertinent to the immediate present case is appropriate when clinical test results are returned, opportunities to include more humanistic elements in these conversations can play a significant role in the cultivation of trust. Multiple studies have shown that the ways in which clinicians communicate with NICU families is critical to promoting or hindering trusting relationships (Labrie et al., 2021; Gallagher et al., 2018). Another study found that attention to humanistic concerns like life experiences “enables the patient to trust the caregiver and the healthcare environment” (Norberg et al., 2017). These more “humanistic” foci—essential to building empathy—have been the subject of bioethics and medical humanities education in medical schools for the past several decades but are seldom translated into specific communication behaviors for clinical conversations.

While the core nature of the type of return-of-results conversations our study analyzed is essentially a task of factually relaying genetic findings, these conversations also influence trust as they represent clinical encounters that reflect on the biomedical enterprise as a whole, as discussed at the outset of this brief report. In this, it would behoove clinicians aiming to cultivate trust among underrepresented populations to include more “humanistic” topics of conversations that recognize the lived experiences and life circumstances of patients.

In terms of specific communication behaviors, numerous studies have shown that acknowledging and expressing concern and empathy for lived experience and life circumstances is key to building trust (Norberg et al., 2017; Rydenlund et al., 2019; Palmér et al., 2024; Dinç and Gastmans, 2013). For example, in addition to the community engagement literature above, literature in both nursing and forensic psychiatry emphasizes the “relational” nature of trust, established through a focus on the whole person and their life circumstances (Dinç and Gastmans, 2013). In nursing, demonstrating respect for patients through “communication, connection, and reciprocity” is identified as essential to establishing trust (Leslie and Lonneman, 2016). In forensic psychiatric care, the hermeneutic approach has emphasized incorporation of life experiences to deepen the caring relationship (Rydenlund et al., 2019). These insights have given rise to a dedicated approach dubbed “lifeworld hermeneutics” which emphasizes “existential phenomena” and life experiences (Norberg et al., 2017; Palmér et al., 2024).

Cultivating trust through specific clinician communication behaviors has been documented among NICU parents caring for children with genetic conditions. A survey of parents caring for children with Trisomy 13 and Trisomy 18 reported that the way in which clinicians communicated information about their child’s genetic condition was key to establishing and maintaining the family’s trust (Janvier et al., 2020). In particular, parents trusted clinicians who shared balanced information about their child’s future, emphasized that their child’s life had value and meaning independent of a potentially life-limiting illness, and confirmed that their child’s diagnosis was not the fault of the parents.

The scarcity of these communion behaviors within our data represents missed opportunities for clinicians to build trusting relationships with families during genetic return of results conversations. The fact that no clinician in our dataset described the “lived experience” of children when explaining the impact of genetic findings on the child’s future health and quality of life may reflect a common bias within the medical community that living with a chronic, medically complex condition is incompatible with a life worth living (Cho et al., 2023). This finding is additionally concerning considering the role this information plays in treatment recommendations and decision-making for infants with genetic diagnoses and disabilities (Callahan et al., 2022; Oslin et al., 2025; Bogetz et al., 2021).

## 5 Conclusion

Attaining the sample diversity necessary for good science among biobank participants will require cultivation of trust with underrepresented populations. Community engagement, especially CBPR approaches, emphasize that trust is best established over time, across a spectrum of encounters, rather than contained within the parameters of single-iteration research or clinical encounters. In this regard, clinical encounters have failed to capitalize on opportunities to promote trust by extending clinical conversations beyond the confines of core concerns of relaying factual information to include more humanistic elements that focus on life circumstances and lived experience of health conditions.

**FIGURE 1:**
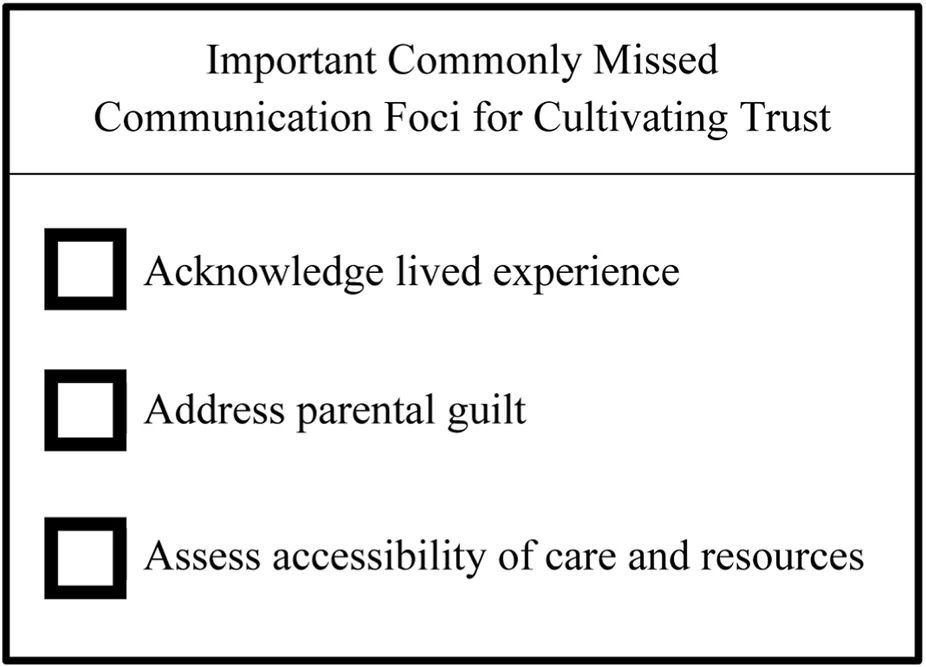
Important commonly missed communication foci for cultivating trust.

Researchers would do well to emphasize communication frameworks that help clinicians prepare for and navigate prognostic, including quality of life, discussions that provide balanced information about the future, incorporate life circumstances, and acknowledge a child’s inherent value (Racine et al., 2017; Lemmon et al., 2023) (See Figure 1).

As discussed above, the importance of humanistic communication behaviors have been identified in our own team’s prior research, which found phenomena tied to lived experience and life circumstances to be identified by members of underrepresented groups as particularly pertinent to trust (May et al., 2021; Smith et al., 2025).

The salience of lived experience is increasingly recognized in a variety of contexts across the globe. Authors of a discussion of trust and lived experience in *The Conversation* describe government agencies increasingly adding community representatives to committees to better understand lived experience (citing in particular a New Zealand Initiative employing peer support workers), and university initiatives seeking to add perspectives representing lived experience to research endeavors (Ahir-Knight and Godfrey, 2026). A Pew Research Center study of trust in 25 countries around the world found gaps in trust according to age, income and education, all central to lived experience (Lippert and Schulman, 2025).

The importance of communication behaviors for trust means that biobank (and other) research initiatives will need to promote strategies of communication cultivating trust–across the spectrum of medical interactions–if data sets are to reflect the sample size and diversity needed for good science. The inclusion of such communication behaviors may help clinicians detect the unexpected challenges and unidentified needs of families caring for critically ill children, as well as express empathy and connection that makes patients and their families feel valued, respected, and “seen” by their medical providers.

## Data Availability

The data analyzed in this study is subject to the following licenses/restrictions: Original Data held under different IRB protocol at the University of Alabama Birmingham. Requests to access these datasets should be directed to Bruce Korf, bkorf@uabmc.edu.
